# MResTNet: A Multi-Resolution Transformer Framework with CNN Extensions for Semantic Segmentation

**DOI:** 10.3390/jimaging10060125

**Published:** 2024-05-21

**Authors:** Nikolaos Detsikas, Nikolaos Mitianoudis, Ioannis Pratikakis

**Affiliations:** Electrical and Computer Engineering Department, Democritus University of Thrace, University Campus Xanthi-Kimmeria, 67100 Xanthi, Greece; ndetsika@ee.duth.gr (N.D.); ipratika@ee.duth.gr (I.P.)

**Keywords:** semantic segmentation, transformer networks, convolutional neural networks, deep learning, scene understanding

## Abstract

A fundamental task in computer vision is the process of differentiation and identification of different objects or entities in a visual scene using semantic segmentation methods. The advancement of transformer networks has surpassed traditional convolutional neural network (CNN) architectures in terms of segmentation performance. The continuous pursuit of optimal performance, with respect to the popular evaluation metric results, has led to very large architectures that require a significant amount of computational power to operate, making them prohibitive for real-time applications, including autonomous driving. In this paper, we propose a model that leverages a visual transformer encoder with a parallel twin decoder, consisting of a visual transformer decoder and a CNN decoder with multi-resolution connections working in parallel. The two decoders are merged with the aid of two trainable CNN blocks, the fuser that combined the information from the two decoders and the scaler that scales the contribution of each decoder. The proposed model achieves state-of-the-art performance on the Cityscapes and ADE20K datasets, maintaining a low-complexity network that can be used in real-time applications.

## 1. Introduction

Semantic segmentation is a computer vision task that classifies each pixel of an image into a predefined set of categories, or “classes”. The assigned pixel labels constitute regions of different semantic categories, essentially forming the shapes of the objects that appear on the image. Autonomous driving, medical imaging, satellite imagery and augmented reality are some of the popular application fields on which semantic segmentation is employed.

Convolutional neural networks with encoder–decoder blocks have been the primary architectural choice for addressing the semantic segmentation task since the emergence of deep learning. As a next step, Google adapted the transformer architecture to computer vision tasks (classification) with the vision transformer (ViT) model [1] and opened the way for a more extensive use of transformers on other visual tasks, including semantic segmentation.

A major concern in the process of producing new state-of-the-art models for the task is the increased computational complexity. The number of parameters has already grown to immense levels, which range from hundreds of millions to even billions. As a consequence, inference on architectures of that magnitude requires many, expensive, and energy-consuming hardware components (GPUs), which renders large-scale real-time applications prohibitive. For example, real-time semantic segmentation for autonomous vehicles cannot be realized, if the inference task requires seconds or hundreds of milliseconds to complete, even on a high-end GPU.

For this reason, research on high-performance yet low-cost architectures is both not only popular but also necessary. Before focusing on low complexity, real-time architectures, we present some top performing convolutional or transformer-based networks, as they have appeared and progressed in recent years, irrespective of the network size. First, PSPNet [2] (Pyramid Scene Parsing Network) is a semantic segmentation model that utilizes pyramid pooling modules to capture contextual information at different scales. It effectively addresses challenges in scene parsing by aggregating global context information and integrating it into the segmentation process. It can utilize various backbone architectures for feature extraction that render the network either real-time or non-real-time. DeepLabV3+ [3] is a DeepLab family network extension with features, such as atrous spatial pyramid pooling (ASPP), encoder–decoder structures, and depthwise separable convolutions for achieving high-quality segmentation results. InternImage [4] is a large-scale CNN that incorporates deformable convolution [5] variants that allow for capturing long-range dependencies with adaptive spatial aggregation. The HSSN framework, introduced in [6], adapts existing segmentation networks to incorporate hierarchy information for improved network learning. It forms a tree-structured hierarchy of the latent class dependencies, representing concepts and relationships, which is used in training for mapping pixels and their classes and in inference for finding the best path from root to leaf in the class hierarchy. Finally, ref. [7] suggests the use of the Supervised Contrastive Segmentation method that combines supervised learning with contrastive learning to achieve better segmentation results. The method can be used to train architectures containing existing base networks for mapping input to image embedding representations and networks for producing segmentation maps over the class space, without interfering with the base networks during inference.

The emergence of transformers [8] has revolutionized deep learning-based classification, especially with the emergence of the Vision Transformer (ViT) [1]. The Swin Transformer [9] is a hierarchical Transformer, whose representation is computed by leveraging shifted windows that offer the ability to model at various scales. SETR [10] is another transformer-based architecture, which employs a standard transformer encoder [8] and different convolutional decoders. The SETR-PUP variation progressively upsamples the encoded information until producing the output segmentation masks, while the SETR-MLA aggregates streams from different decoder layers. Segmenter [11] is a Vision Transformer (ViT) [1]-based semantic segmentation model, with a standard ViT encoder and either a point-wise linear or a transformer (multi-head self-attention)-based decoder. Several model scales are presented, ranging from tiny to large, with varying performance and complexity, with the tiny architectures being suitable for real-time inference. The SegFormer model [12] consists of a hierarchical Transformer encoder that generates high-resolution coarse features and low-resolution fine features and an MLP decoder for fusing the multi-level information. It uses smaller (4×4) sized patches compared to the standard ViT encoder patches (16×16) and sequences reduced self-attention blocks for faster yet efficient processing. The model is constructed at different scales from MiT-B0 to MiT-B5, with MiT-B0 being small enough for real-time applications. BiSeNet [13] is a versatile semantic segmentation model that offers a good trade-off between efficiency and accuracy, making it well suited for various computer vision tasks, where real-time performance is crucial. It consists of two separate pathways, one for capturing global contextual information and one for fine-grained spatial details, which are combined with the bilateral fusion module. SwiftNetRN-18 [14] is a SwiftNet variation that employs a ResNet-18 backbone along with SwiftNet family features, such as depthwise separable convolutions, channel attention mechanisms, and feature fusion modules, in order to achieve accurate semantic segmentation with real-time efficiency. DDRNet [15] (dilated dense residual network) is another convolutional neural network architecture for semantic segmentation that uses dilated convolutions, residual blocks with dense connections, where each layer is connected to every other layer in a feed-forward manner and hierarchical feature fusion mechanisms that capture multi-scale information by combining features from different network layers. The authors present versions of the architecture with varying levels of complexity, one of which, the DDRNet-23-slim, qualifies for accurate real-time inference. LETNet [16] is a CNN encoder–decoder architecture with a transformer-based bottleneck block and skip connections between the encoder and decoder. The authors also propose the lightweight dilated bottleneck (LDB), a block positioned in the encoder and decoder parts, which is a series of convolutional layers, with residual connections, channel attention, and shuffling mechanisms for collecting more feature information while keeping the number of layers low. RegSeg [17] is based on SE-ResNeXt architectural blocks, which use dilated convolutions for a larger effective field-of-view and optimize the dilation rates with gradient descent applied over a differentiable neural architecture search method. PIDNet [18] is another convolutional approach focusing on real-time performance. The architecture consists of three branches (P, I, and D), aiming to extract high-resolution feature maps, long-range dependencies, and boundary regions, respectively, which are aggregated with multistage convolutional architectural blocks until producing the segmented feature maps. CLUSTSEG [19] views the image segmentation tasks as pixel clustering problems. The proposed framework applies a recursive clustering procedure with a transformer-based cross-attention acting as a cluster solver where queries are considered cluster centers. A different approach, based on prototype-based classification, is adopted by [20]. The authors suggest a model that can be based on either CNN or transformer backbones, where a set of non-learnable class prototypes are used for making dense predictions. Segmentation is achieved by pixel-wise prediction based on distance between pixels and class prototypes.

In this paper, we present a low-cost deep learning architecture, featuring transformer and CNN architectural features, that combines relatively a low computational cost with a robust semantic segmentation performance. The novel offerings are:

- A novel low-cost real-time architecture based on transformers and CNN that achieves the best performance among the state-of-the-art peers.
- A complete ablation study that explores the offerings of convolutional blocks in a transformer framework.
- The introduction of a multi-resolution framework in a transformer-based architecture.
- Automated tuning of hyper-parameters using specialized networks, such as the fuser and the scaler.

An outline of the paper is given as follows. In Section 2, the novel architecture MResTNet is presented. In Section 3, we ablate over a number of architectural options in order to determine the optimal structure of the proposed network. In Section 4, the final results on the Cityscapes and ADE20K datasets are presented. Section 5 concludes the paper summarizing the offerings.

## 2. The Proposed MResTNet

**Figure 1:**
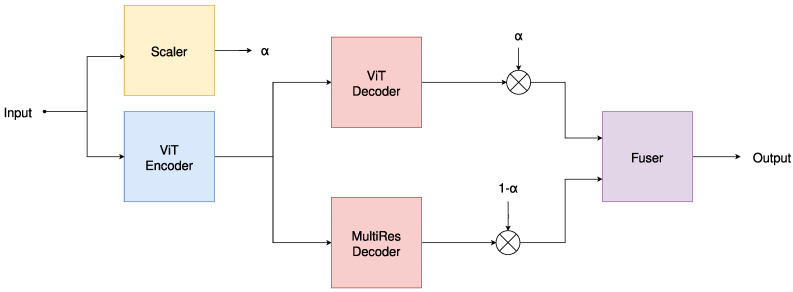
The proposed MResTNet architecture uses a ViT encoder and decoder, along with a convolutional multi-resolution decoder and two networks (scaler and fuser) that estimate a method hyperparameter (scaler) and combine the two decoders (fuser).

The proposed architecture is primarily an encoder–decoder model influenced from both transformer and convolutional architectures. It leverages scalability, parameter efficiency, and improved performance relative to the architecture scale. The proposed architecture is outlined in Figure 1.

By focusing on architectural modifications on the decoder side, rather than the encoder, we circumvent the need for extensive pre-training of a new encoder architecture. This strategic choice helps us sidestep significant overhead and computational expenses associated with training large-scale models from scratch. By prioritizing changes in the decoder, we can efficiently leverage the learned representations from existing pre-trained encoders, thereby streamlining the experimentation process and accelerating model development. Moreover, this approach allows us to iteratively refine and optimize the segmentation model architecture, leading to quicker iterations and the more efficient utilization of computational resources. The benefit of using available pre-trained ViT models to various downstream tasks has also been emphasized in [21].

### 2.1. Encoder

**Figure 2:**
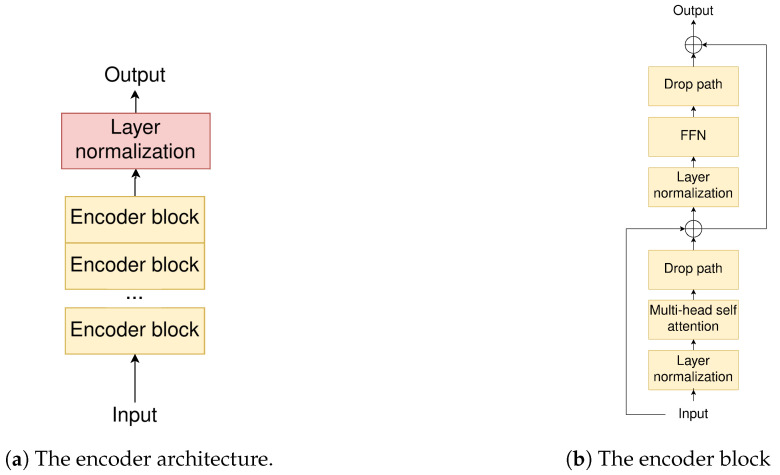
The proposed ViT encoder architecture used in MResTNet.

The encoder (Figure 2a) is a ViT encoder [1] that offers improved global context modeling by capturing long-range dependencies across the entire image without the convolutional layer complexity that would have been otherwise necessary.

The encoder was pre-trained on the ImageNet-21k dataset, according to [22], with regularization and data augmentation. The concept of pre-training and transferring to downstream tasks has also been extensively studied in [23], where pre-training on even larger-scale upstream tasks has been investigated.

We summarize here the standard ViT encoder process [1], which our encoder follows. The image is split into non-overlapping 16×16 patches through a 2D convolution, which also extends the number of filters to the chosen model dimensionality (192). The spatial dimensions of the resulting feature map are then flattened and a trainable class token of the same dimensionality is added. Finally, trainable positional embeddings are added, to capture the spatial information, that would otherwise be lost due to the nature of the transformer inductive bias (positional agnostic). The encoder then sequentially applies a number (12) of encoder blocks (Figure 2b), with each block normalizing its input and applying multi-head self-attention. The result is finally added to the input, using a residual connection, and normalized again before passing through a feed-forward network, with a final residual connection before the output. A drop path regularization is added before each residual connection. The encoder output is finally normalized and forwarded to the decoding stage.

### 2.2. Decoder

**Figure 3:**
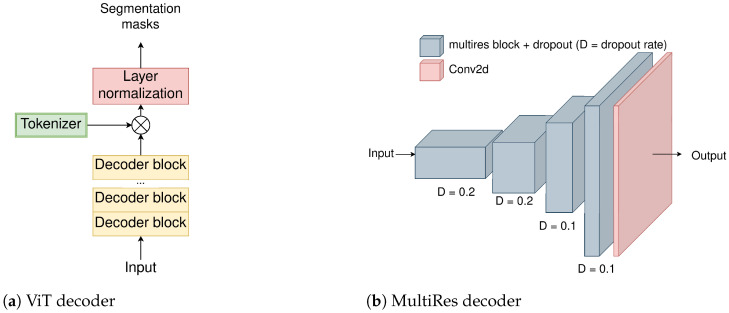
The decoder of the proposed MResTNet architecture comprising the ViT decoder and the MultiRes decoder.

The decoder consists of a ViT [1] and a multi-resolution (MultiRes) decoder in parallel. The ViT decoder (Figure 3a) applies a series of decoder blocks (2) and reduces the output dimensionality to the number of the dataset classes by multiplying the output with a trainable matrix, named “Tokenizer”, before normalizing the result. The Tokenizer matrix is of dimension DxK where *D* is the model dimensionality and *K* is the number of classes. The decoder blocks operate in the same manner as the blocks of the encoder. When the decoding is completed, the output is a 1D vector, as is in the case of Transformer decoders, which is then rearranged to a 2D shape. In addition to that, since in the beginning of the information flow in the Transformer encoder–decoder architecture, the input image is subject to patching, the resulting shape is not the same as that of the input image or the ground truth segmentation masks. For example, as in the case of Cityscapes dataset, the input images of shape 768×768 are patched to the feature maps of shape 48×48. The feature maps must be upscaled to the proper dimensions. In our case, this is performed using bilinear interpolation.

**Figure 4:**
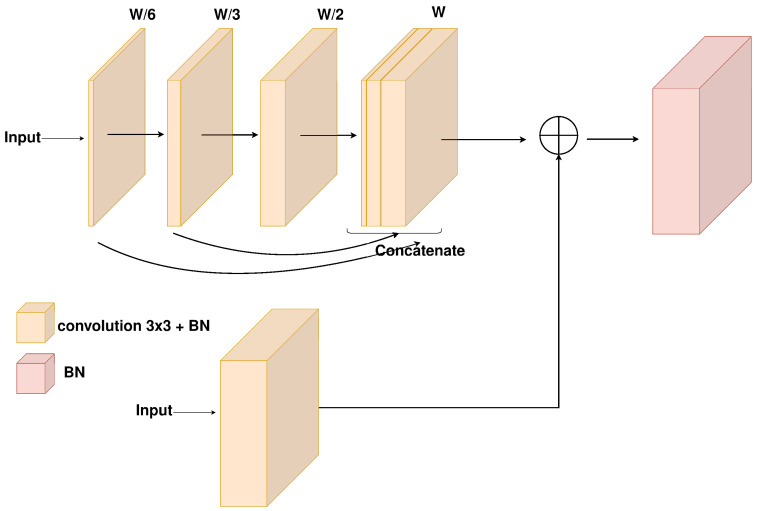
The MultiRes block inside the MultiRes decoder.

The MultiRes decoder (Figure 3b) consists of a series of spatially upscaling and channel contracting MultiRes blocks (Figure 4) that aim to provide the spatial information that was lost during the encoding process and refine the segmentation predictions. As Ibtehaz and Rahman propose in [24], the MultiRes block is a replacement of Inception blocks without any of the increased complexity of the larger (5×5 and 7×7) convolutions. As also shown in [25], Detsikas et al. supported the improved performance of the MultiRes block, with a comparatively insignificant overhead, and extended experimentation in the document image binarization task.

### 2.3. Aggregation

**Figure 5:**
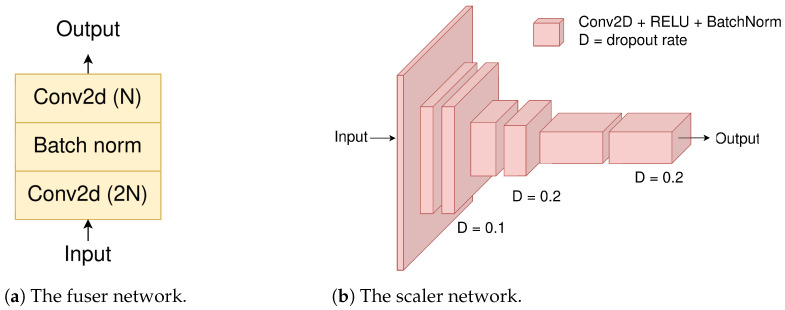
The decoding aggregation modules.

The two decoder outputs are aggregated, employing the scaling and fusing blocks. The scaler (Figure 5a) produces a single scalar factor for each patch by gradually reducing the input spatial dimensions through convolutional layers. The scaling factor α (and 1−α, respectively) acts as a balancing weight between the two decoder outputs. The reason for not using a separate scaling block for each decoder output is to avoid excessive network complexity. As seen in Table 1, the scaler adds 0.2 M parameters. In addition to that, we also aim to induce a complementary character to the two decoder outputs, which reflects the natural complementary benefits offered by the Transformer- and CNN-based architectures (transformers better model long range dependencies and capture global context, while CNNs better capture spatial information and local patterns and structures). The network consists of several 2D-convolutional layers that feature the ReLU activation, batch normalization [26], and dropout [27] with a ratio denoted by variable *D*.

On the other hand, the Fuser block (Figure 5a) is a convolutional module that merges the concatenated scaled decoder outputs and produces the final output segmentation maps. The module consists of a 2D convolutional layer, a batch normalization layer, and a final 2D convolutional layer.

Both aggregation blocks operate on feature maps with restored 2D spatial dimensions and channel depth equal to the number of classes. In other words, each decoder output is a representation that no longer contains encoded information. The aggregation blocks are a mechanism of fine tuning the decoded information in order to exploit the benefits of the Transformer and CNN worlds. The transformer decoder recovers the encoded information, exploiting the long-range dependencies captured from the ViT encoder, while the MultiRes (CNN) decoder restores the spatial information lost during the encoding.

## 3. Ablation Study

The design choices and architectural components of a semantic segmentation model significantly impact the performance and the generalization ability of the network. In this study, we systematically analyze the contributions of various components within our semantic model. We aim to gain insights into the effect of individual design choices in terms of performance and network computational complexity.

This study was conducted over the Cityscapes dataset [28] and the networks were trained for 216 epochs, employing the SGD optimizer, with a polynomial decay rate, initialized at 0.01, of power 0.9, decaying at every step. The final selected model was trained for 864 epochs, with the same optimizer and learning rate parameters, with the exception of the decaying step size, which was set to 4. The model was developed in Python 3.10.12 using PyTorch 1.7.1 on a PC with i9-11900F, 64 GB RAM, an NVidia RTX A6000 GPU 48 GB, running Ubuntu Linux 22.04.

The baseline model for the study is an encoder–decoder model containing only the described ViT encoder and transformer decoder parts. First, we studied several architectural variants by adding a parallel MultiRes decoding path to the baseline model and extending it with the fuser and scaler blocks. We also experimented with the scaler architecture, since it allowed for several options that significantly affected the model performance. We then studied the effect of the loss function on the model training. We used a cross-entropy loss as well as a combination of cross-entropy and dice loss. Finally, in an attempt to create the smallest possible model that would maintain a high prediction performance, with respect to the dominant metrics of the field (mean intersection over union—mIoU), in comparison to other SOTA methods, we created and evaluated significantly smaller models.

### 3.1. Architectural Variants

Our baseline model is a ViT encoder–decoder model, with the previously described encoder and decoder. We gradually added MultiRes blocks, scaler, and fuser to evaluate performance against complexity scaling. For evaluating architectures without Scaler and Fuser, we simply added the two parallel decoded outputs.

As depicted in Table 1, each of these blocks gradually improves the performance, without large model-size overhead. In particular, the fuser and the scaler have a relatively insignificant size impact. Our primary goal is to extend the decoding capability of the model’s decoder side. We focus on the decoder side, since we do not need pre-training, while encoder side changes are not treated fairly without pre-training.

We firstly extend the existing decoder with more decoder blocks. The baseline decoder has two blocks and we add two more. It is natural to assume that the extra network decoder capacity alone, due to the additional blocks, would be enough to improve the output accuracy metrics. If more network capacity was the only target, simply adding ViT decoder layers with the same additional parameters would still bring performance improvements. As seen in Table 1, this “extended decoder” architecture does not yield any significant improvements, while the “dual decoder” does. It is reasonable to deduce that the convolutional decoder preserves and restores the encoded spatial information and feature arrangement, while their inherent gradual upscaling operation recovers fine details.

Replacing the standard decoder convolutions with MultiRes blocks introduces yet another significant performance improvement, considering the scale of the model. By processing multiple resolutions in a hierarchical manner, MultiRes blocks can decode both fine-grained details and coarse contextual information more efficiently. Table 1 supports this upgrade, by showing the number of parameters and the mean intersection over union (mIoU) (semantic segmentation quality index).

Summarizing all the above, so far, we evaluated the dual decoder against the single transformer decoder, but not against the single convolutional decoder. However, in order to establish the necessity of both decoders, we have to evaluate this dual-model against the convolutional decoder alone. The “convolutional decoder” of Table 1 shows exactly that it yields no improvement. The Transformer decoder cannot be left out of the model, since it incorporates global context into the segmentation process more effectively, since transformers excel at capturing long-range dependencies between pixels. Semantic segmentation does require capturing contextual information from the distant regions of the input image to accurately classify each pixel.

The power of the two approaches can be better harvested with the fuser and scaler blocks. Looking at Table 1, it is clear that the Fuser improves the performance. The fuser block (Figure 5a) convolutionally merges the two decoded outputs.

The scaler block increases even more the accuracy metric (mIoU), since it acts as a balancing factor between the decoded outputs, selecting the best elements each one has to offer in the overall decoding. The scaler produces a 2D feature map that has the same size as that of the input after patching has been applied. The scaler output values end up in the range of 0.6–0.7, with a mean value of ∼0.65. This shows that the scaler output is neither too small (∼0) nor too high (∼1), cases that would have rendered the use of one of the two decoders unnecessary.

In Table 2, we compare the effect of using the actual predicted scaler output against using fixed scaler values. We make our evaluation on the Cityscapes validation set. The first column shows the result of using the scale produced by the scaler, while for all the other columns, a fixed scaler value was used instead. The experiment clearly shows that the predicted scaler output produces better results by appropriately scaling both decoders. Setting a fixed value produces sub-optimal results and degrades the performance.

**Figure 6:**
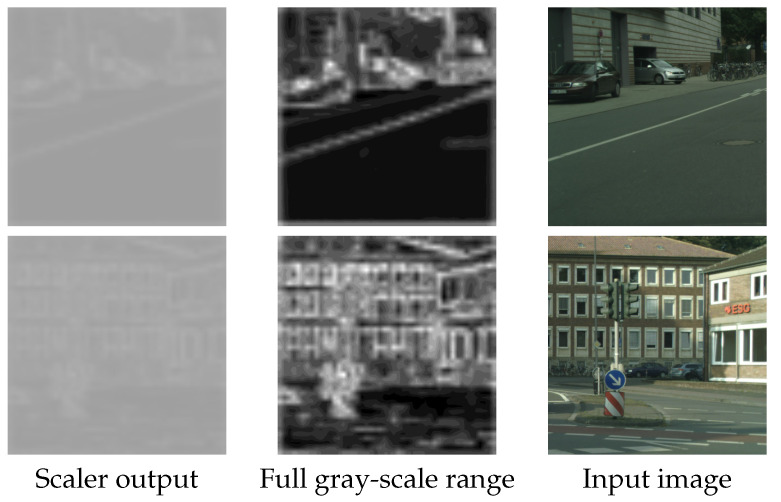
Resized scaler output visualization for various images. The first column shows the actual scaler output, the second column shows the output normalized for better visualization, and the third is the input image.

In addition, Figure 6 visualizes the scaler output, resized to the original image size (the actual scaler output size is the same as that after input patching, which in this case is 48×48). The first column shows the actual output, while in the second, the values are linearly normalized to [0,255] for better visualization. The final column is the input image. The scaler acts differently on image features and it is evident that edge information receives higher scaler values, for the transformer decoder.

### 3.2. Scaler Variants

As seen in Table 1, the use of fuser and scaler enhances the performance, without any significant complexity overhead. We can investigate further alternative structures for these blocks in order to experiment with further performance improvements. The Fuser does not leave much room for investigation, since it is a block that is applied to the decoded 2D masks. The simplest and most obvious choice is the one already made. However, the Scaler, which is applied to the input, or in a more abstract description, to the information before the encoding, does allow for architectural experimentation.

**Figure 7:**
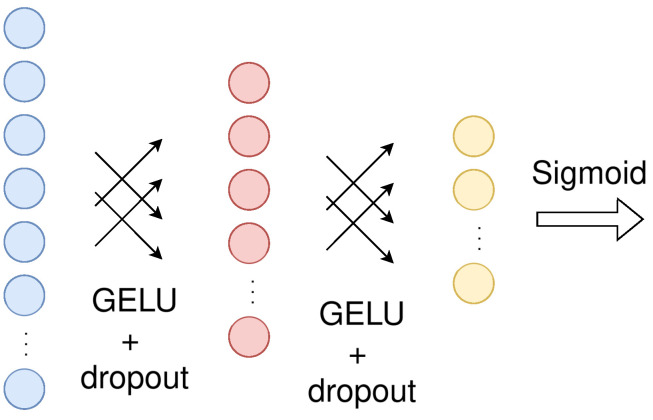
The proposed feed-forward scaler block.

The Scaler design (Figure 5b), that was described earlier on, can be replaced by a feed-forward network (Figure 7). Applying the scaler to the flattened 2D input would require a very large feed-forward network, so it is more appropriate to apply it to the patch-embedded input, which is already flattened and much smaller in dimension. Since encoding can benefit from the positional embeddings (lost inductive bias), we can assume that using the positional embeddings in the scaler block would be beneficial as well. Consequently, we gather that we can apply the feed-forward scaler after the positional embedding addition, just before the encoding. The feed-forward scaler consists of three dense contracting layers, which reduce the dimensionality to a single scaler value that later scales the decoded outputs.

The results are depicted in Table 3. The complexity shown in the Table reflects the whole model, incorporating the respective scaler. The CNN scaler performs better, with an overhead that we can afford to spend.

### 3.3. Loss Function Ablation

We have trained our model using the cross-entropy loss and with a weighted combination of cross-entropy and dice loss. The dice loss alone proved incapable of providing considerable results.

The cross-entropy loss is a standard for classification problems with multiple classes

(1) $$L_{\mathrm{CE}}=-\frac{1}{N}\sum_{i=1}^{N}\sum_{c=1}^{C}y_{i,c}\log\left({\hat{y}}_{i,c}\right)$$

where

- $L_{\mathrm{CE}}$ is the cross-entropy loss.
- *N* is the total number of pixels.
- *C* is the number of classes.
- $y_{i,c}$ is the ground truth label (1 if the pixel belongs to class *c*, 0 otherwise) for pixel *i* and class *c*.
- ${\hat{y}}_{i,c}$ is the predicted probability of pixel *i* belonging to class *c*.

The Dice loss for multiple classes is formulated as follows:

(2) $$\mathrm{Dice}\;\mathrm{Loss}=1-\frac{2\sum_{i=1}^{N}y_{i}{\hat{y}}_{i}}{\sum_{i=1}^{N}y_{i}+\sum_{i=1}^{N}{\hat{y}}_{i}}$$

where:

- *N* is the total number of pixels.
- $y_{i}$ is the ground truth label for pixel *i*.
- ${\hat{y}}_{i}$ is the predicted label for pixel *i*.

The dice loss usually alleviates the class imbalances present in the data. In the datasets used in our experiments, not all classes are equally represented. Thus, we would have expected that employing the dice loss would improve performance. Unfortunately, this did not happen in our simulations (see Table 4). Most likely, the answer is related to the fact that the dice loss itself is not as smooth as other loss functions, such as cross-entropy, an issue that becomes more serious in multiple-class classification problems. Optimizing the loss function in such a scenario is more challenging, especially when using gradient-based methods, such as stochastic gradient descent (SGD), which is used in our experiments.

### 3.4. Model Size Ablation

Table 5 depicts the complexity of each architectural block used. It is clear that the encoder is the dominant part in terms of complexity and it is the ideal candidate for intervention in order to scale down the model. For this reason, we trained a scaled-down version of the model, using 8 instead of 12 encoder blocks.

Table 6 shows the results of scaling the model down. This smaller model presents a direction of further improving an even more scaled-down model. Nonetheless, in the context of this study, we proceed with the larger of the two in order not to lose performance.

## 4. Experimental Results

Once we confirmed the final proposed architecture of MResTNet, we evaluated our model against other state-of-the-art methods that also have tiny scale variants for two popular semantic segmentation datasets: the Cityscapes [28] and the ADE20K [29]. The proposed MResTNet is implemented in Python 3.10.12 using PyTorch 1.7.1 and is publicly available (https://github.com/detsikas/MResTNet, accessed on 20 May 2024).

### 4.1. Cityscapes

Cityscapes is a semantic segmentation dataset consisting of 5000 fine-annotated, high-resolution images with 19 categories in urban driving environments. The dataset consists of 2975 training, 500 validation, and 1525 test images.

In our comparison, most of the referenced methods have variants in multiple complexity scales. There are models with billions of parameters, models with hundred millions of parameters, and some models with some millions of parameters that are usually referred to as real-time variants [12]. For example, DeepLabV3+ also has a variant, based on ResNet-101, with 62.7 million parameters, while the model we compare here has only 15.4 and is based on MobileNetV2. In these cases, we only consider the real-time variants, since the aim of the proposed architecture is to build an efficient, real-time model. In our comparison, we also omitted methods that indeed have a top metric performance, but also have a complexity that is far from the real time domain (>15 M). Such methods are the InternImage-H [4] with 1.08 B parameters and 87.0 MIoU and the SETR [10] with 318.3 M parameters and 82.2 MIoU.

Table 7 shows the comparison results on the Cityscapes dataset with respect to other SOTA methods of real-time scale (up to ∼15 M parameters). The methods are compared in terms of the number of parameters, floating point operations per second (GFLOPS), and mean intersection over union (MIoU) for classification accuracy. MResTNet ranks first in the MIoU metric among all the methods, while at the same time, it maintains a very low and well within real-time constraints calculation complexity, as shown in both the number of model parameters and floating point operations at the inference time. LETNet [16] is the most light-weight from the compared methods, but its performance is far inferior to the other methods. A very close runner-up to the proposed method is RegSeg [17], which has a lot fewer number of parameters, but a slightly higher number of GFLOPS and a slightly inferior classification performance.

**Figure 8:**
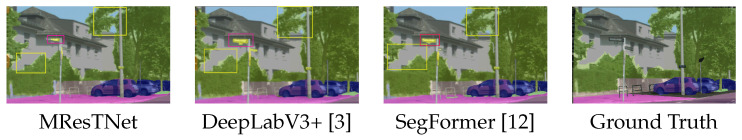
Comparison with SegFormer and DeepLabV3+ on the Cityscapes dataset. Yellow rectangles show regions where our method predicts more precise region outlines while the red rectangle shows an area that was partially filled. Different colors show different classes.

**Figure 9:**
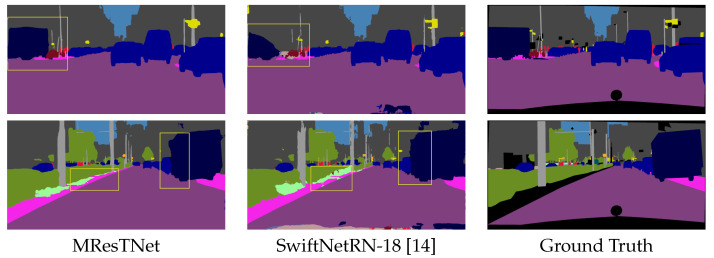
Comparison with SwiftNetRN-18 on the Cityscapes dataset. The proposed method captures masks with more accurate boundaries and exhibits less false detections and noise as shown by the yellow rectangles. Different colors show different classes.

**Figure 10:**
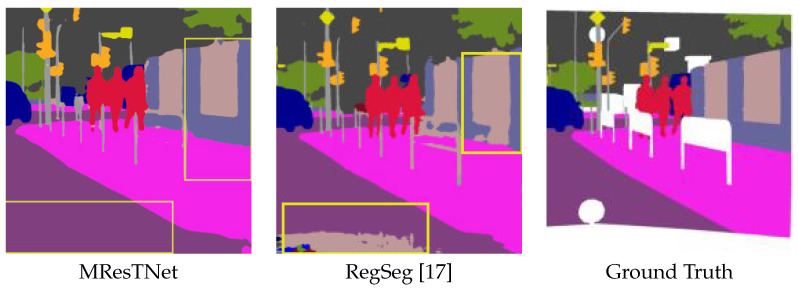
Comparison with SegFormer and RegSeg on the Cityscapes dataset. Yellow rectangles show areas where our method produces less false detections and smoother regions. Different colors show different classes.

Figure 8, Figure 9 and Figure 10 show comparisons of visual semantic segmentation results between the proposed MResTNet and other runner-up methods. It can be noticed that MResTNet produces clearer and more distinct area boundaries with fewer errors in detection.

### 4.2. ADE20K

The ADE20K dataset contains densely annotated images covering a wide range of indoor and outdoor scenes covering 150 object classes and consisting of 20,210 training images, 2000 images for validation, and 3352 images for testing.

**Figure 11:**
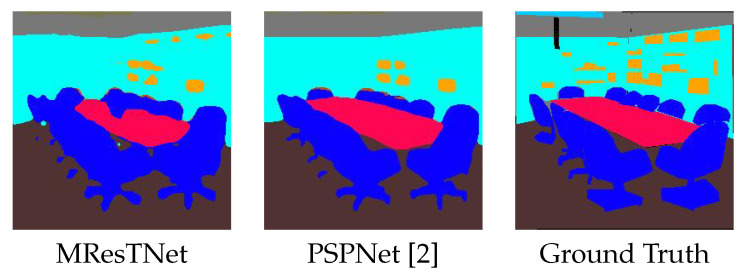
Comparison with PSPNet on the ADE20K dataset. PSPNet detects clearer boundaries but MResTNet detects considerably more objects. Different colors show different classes.

**Figure 12:**
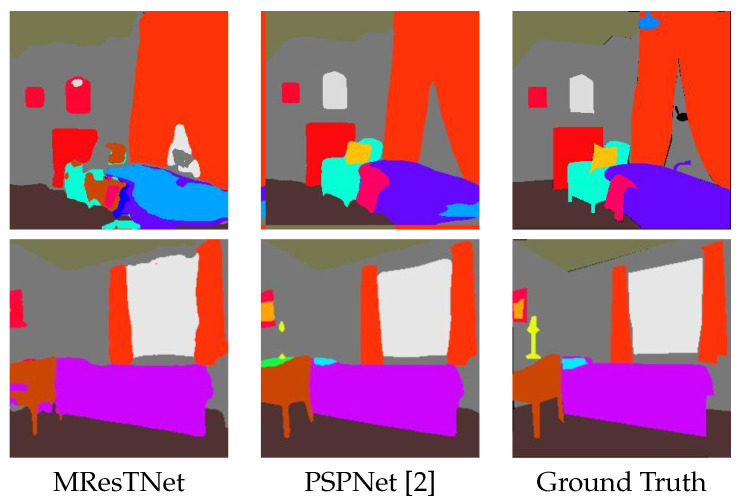
Inference quality degradation (against PSPNet) on the ADE20K dataset. Different colors show different classes.

Table 8 shows the comparison results on the ADE20K dataset with respect to other state-of-the-art methods of real-time scale. Again, MResTNet ranks first in mIoU metric among all the methods with a small computational complexity, well below the real-time boundary. This shows that the proposed MResTNet architecture is a robust semantic segmentation architecture that can operate efficiently on other datasets/applications, apart from Cityscapes. Figure 11 shows indicative comparison results of MResTNet with other SOTA methods evaluated on the ADE20K datasets, while Figure 12 focuses on the inference quality degradation that is sometimes exhibited by MResTNet on ADE20K dataset. Overall, our method is comparable to the rest of the top ranked methods, detecting more objects with occasionally less-precise object boundaries.

## 5. Conclusions

In this paper, we presented a lightweight and efficient architecture, the MResTNet, for performing semantic segmentation. Through systematic experimentation and ablation study, we observed several key findings that led to the formation of the proposed model. Firstly, we observed that the inclusion of an additional parallel MultiRes decoder, which incorporates an extracting convolutional decoding operation with the use of MultiRes blocks, significantly improves the segmentation efficiency. Additionally, we observed that the introduction of aggregation blocks effectively combines the benefits of both Transformer and CNN domains, leading to an insignificantly better model performance in terms of the real-time efficiency of the architectural overhead. The proposed model features the best performance compared to other SOTA models with similar number of parameters and GFLOPS, which demonstrates that it can perform well both in terms of performance and real-time implementation.

In general, this study provides valuable insights into improving the transformer semantic segmentation model performance with the CNN architectural blocks, while prioritizing the compact network size suitable for real-time applications.

## Figures and Tables

**Table 1:** Architectural variants of the proposed architecture determine the optimal architectural selection (performed on Cityscapes dataset).

Variant	Parameters	Mean IoU
Baseline	6.2 M	72.29
Extended decoder	6.7 M	72.44
Convolutional decoder	6.1 M	56.99
Dual decoder	6.3 M	72.93
Dual MultiRes decoder	6.5 M	73.18
Dual decoder with fuser	6.5 M	73.3
Dual decoder with scaler and fuser	6.7 M	73.59

**Table 2:** Scaler impact on mIoU for different fixed scaler values (Cityscapes dataset). Scaler(I) is the output of the scaler when applied to the input image. The remaining values are arbitrary constant values that replace the scaler predicted value.

α=Scaler(I)	α=0.1	α=0.2	α=0.3	α=0.4	α=0.5	α=0.6	α=0.7	α=0.8	α=0.9
73.59	13.73	26.27	47.7	63.22	70.17	72.27	72.92	72.81	72.17

**Table 3:** Scaler variants ablation (studied on the Cityscapes dataset).

Variant	Parameters	mIoU
(Convolutional) scaler	6.7 M	73.59
Feed-forwardscaler	6.5 M	62.67

**Table 4:** Loss function ablation on the Cityscapes dataset. Optimization does not benefit from any combination of cross-entropy with the dice loss.

Loss	Mean IoU
Cross-entropy	73.59
Cross-entropy + Dice Loss	73.32
Cross-entropy + 0.2 × Dice Loss	73.02

**Table 5:** Architectural blocks complexity (studied on Cityscapes dataset).

Block	Parameters
Encoder	5,338,752
ViT decoder	893,414
MultiRes decoder	251,471
Scaler	292,257
Fuser	13,851

**Table 6:** Model size ablation on Cityscapes dataset.

Model	Mean IoU
Proposed MResTNet (12 enc blocks)	73.59
8 enc blocks	71.9

**Table 7:** Comparison on the Cityscapes dataset.

Method	Parameters	GFLOPS	MIoU
PSPNet (MobileNetV2) [2]	13. 7 M ^1^	423.4 ^1^	70.2 ^1^
DeepLabV3+ (MobileNetV2) [3]	15.4 M ^1^	555.4 ^1^	75.2 ^1^
SegFormer [12]	3.8 M	125.5	76.2
Segmenter [11]	7.1 M	38.54	72.66
BiSeNet [13]	5.8 M	14.8 (at 1536 × 768)	68.4
SwiftNetRN-18 [14]	11.8 M	104	75.5
LETNet [16]	0.95 M	13.6	72.8
RegSeg [17]	3.34 M	39.1	78.5
PIDNet [18]	7.6 M	46.3	78.8
MResTNet	6.79 M	38.44	78.89

^1^ Source: [12].

**Table 8:** Comparison on the ADE20K dataset.

Method	Parameters	GFLOPS	MIoU
PSPNet (MobileNetV2) [2]	13.7 M ^1^	52.9 ^1^	29.6 ^1^
DeepLabV3+ (MobileNetV2) [3]	15.4 M ^1^	69.4 ^1^	34.0 ^1^
SegFormer [12]	3.8 M	8.4	37.4
Segmenter [11]	7.1 M	6.72	38.8
MResTNet	6.79 M	17.09	39.19

^1^ Source: [12].

## Data Availability

The data used in this paper are publicly available from their creators.
